# Analysis of differentially expressed genes related to acute lung injury and their role in metabolic pathways: An integrative study using GEO data

**DOI:** 10.1097/MD.0000000000048519

**Published:** 2026-05-01

**Authors:** Yao Yang

**Affiliations:** a Zhenjiang Integrated Traditional Chinese and Western Medicine Hospital, Zhenjiang, Jiangsu Province, China.

**Keywords:** acute lung injury, biomarkers, differentially expressed genes, mitochondrial energy metabolism

## Abstract

Acute lung injury (ALI) is a life-threatening pulmonary disorder with high morbidity and mortality, and current treatments remain limited. Mitochondrial energy metabolism plays a key role in ALI pathophysiology. This research aims to systematically explore the relationship between mitochondrial energy metabolism and ALI pathogenesis, thereby advancing our understanding of the condition and informing the establishment of improved treatment strategies. In this study, we systematically investigated its involvement through comprehensive bioinformatics analyses of publicly available Gene Expression Omnibus datasets, including differential expression analysis, functional enrichment, immune infiltration profiling, protein-protein interaction network construction, and regulatory network prediction, with the aim of elucidating disease mechanisms and identifying potential therapeutic targets. Differential expression analysis identified 575 differentially expressed genes (DEGs), comprising 431 and 144 upregulated and downregulated genes, respectively. Subsequent pathway analysis revealed that mitochondrial energy metabolism-related DEGs were significantly enriched in fatty acid oxidation and other key metabolic processes, highlighting the crucial role of mitochondrial dysfunction in ALI pathogenesis. Additionally, immune cell infiltration analysis indicated obvious differences in the composition of 11 immune cell types between ALI and control samples (*P* < .05), suggesting potential avenues for immunotherapeutic interventions. The protein-protein interaction network identified 12 mitochondrial energy metabolism-related DEGs with significant connectivity, from which 9 hub genes were prioritized as promising therapeutic targets. Furthermore, regulatory network analyses elucidated interactions among these hub genes, transcription factors, and miRNAs. Despite limitations, such as the absence of experimental validation and the potential influence of batch effects, this study provides new insights into the molecular mechanisms of ALI and establishes a foundation for future research on metabolic modulation and personalized therapeutic strategies to improve patient outcomes.

## 1. Introduction

Acute lung injury (ALI) is a major clinical concern, defined by rapid-onset respiratory insufficiency and substantial health impacts. ALI can be induced by diverse insults, including trauma, sepsis, and pneumonia, reflecting its complex and multifactorial etiology. The impact of ALI extends beyond individual patients, placing a considerable financial and logistical burden on healthcare systems due to prolonged hospitalizations, intensive care requirements, and high treatment costs.^[1]^ Despite advances in elucidating the pathophysiological mechanisms underlying ALI, therapeutic options remain largely limited to supportive care measures, such as mechanical ventilation (MV) and fluid management.^[2]^ The absence of effective targeted therapies continues to hinder improvements in patient outcomes and contributes to variability in treatment responses.^[3]^

Inflammation and oxidative stress have long been recognized as central drivers of ALI pathogenesis. More recently, mitochondrial dysfunction has emerged as a critical contributor to the onset and progression of lung injury.^[1]^ As key regulators of cellular energy metabolism, mitochondria are essential for maintaining homeostasis, and their impairment can disrupt energy production, exacerbate oxidative damage, and increase cellular vulnerability to injury. Experimental studies have shown that circulating mitochondrial DNA can aggravate ALI by activating the STING pathway and impairing autophagy.^[2]^ Growing evidence indicates that disturbances in mitochondrial energy metabolism play a decisive role in shaping both the severity and clinical outcomes of ALI, positioning mitochondrial pathways as promising therapeutic targets.^[3]^ Nonetheless, the specific mitochondrial genes and metabolic circuits involved remain poorly defined, emphasizing the need for systematic investigation to clarify their roles and therapeutic potential.

To address this knowledge gap, our study investigates mitochondrial energy metabolism as a key phenotypic factor associated with ALI. By utilizing integrated bioinformatics approaches, including differential gene expression and pathway enrichment analyses, we aim to identify mitochondrial energy metabolism-related differentially expressed genes (MEMRDEGs) in ALI. These computational methods enable the interrogation of large-scale genomic datasets, offering critical insights into the underlying biology that can inform the development of targeted therapies.^[4]^ By clarifying the specific roles and functional significance of MEMRDEGs, this study aims to identify novel biomarkers and potential therapeutic targets for the management of ALI while also laying the groundwork for future investigations into the complex interplay between mitochondrial dysfunction and disease progression.^[1,3]^ These findings are expected to advance ALI research and support the development of more effective clinical interventions.

## 2. Materials and methods

### 2.1. Data acquisition

Gene expression profiles were retrieved from the Gene Expression Omnibus (GEO) database (https://www.ncbi.nlm.nih.gov/geo/) using GEOquery.^[5,6]^ Two ALI-related datasets, GSE102016 and GSE2411, were selected for analysis. Both datasets consist of samples derived from *Mus musculus* lung tissue.^[7,8]^ Detailed microarray platform and sample information for these datasets are summarized in Table 1. The microarray platform for GSE102016 is GPL20837, whereas GSE2411 is based on the GPL339 platform. GSE102016 is a lipopolysaccharide (LPS)-induced mouse ALI model that has been widely used to simulate the pathological process of inflammation-related ALI and thus serves as the reference for defining ALI samples in this study.^[9]^ GSE102016 includes 4 ALI samples, 3 healthy control samples, and 8 samples related to lung cancer. The GSE2411 dataset includes control, MV, LPS, and MV+LPS treatment conditions. This study focuses on ALI driven by inflammatory responses, defining the LPS-treated group as the ALI group and including the control group in the analysis. MV-related treatments involve additional mechanical stress factors, resulting in molecular alterations distinct from those seen in pure inflammatory ALI. Therefore, the MV+LPS group was excluded from this meta-analysis to reduce model heterogeneity during cross-dataset integration.

**Table 1:** GEO microarray chip information.

	GSE102016	GSE2411
Platform	GPL20837	GPL339
Species	*Mus musculus*	*Mus musculus*
Tissue	Lung	Lung
Samples in ALI group	4	6
Samples in control group	3	6
Reference	PMID: 28987381	PMID: 16116230

ALI = acute lung injury, GEO = Gene Expression Omnibus.

To identify MEMRDEGs, 2 complementary approaches were used. First, we retrieved relevant genes from GeneCards (https://www.genecards.org/) by searching for the term “Mitochondrial Energy Metabolism.” Genes encoding proteins were exclusively retained, yielding 221 genes.^[10]^ Second, a comprehensive PubMed search was performed using the same search term (focus on studies that provide systematic summaries of mitochondrial energy metabolism pathways, fatty acid oxidation, and mitochondrial function regulation in the literature), resulting in an additional 188 genes.^[11]^ Following merging and deduplication, 386 unique human mitochondrial energy metabolism-related genes (MEMRGs) were identified. Subsequently, these genes were mapped to mouse homologs using the homologene package, producing a final set of 351 mouse MEMRGs. Details of the MEMRG list are provided in Table S1, Supplemental Digital Content, https://links.lww.com/MD/R782.

**Figure 1.**
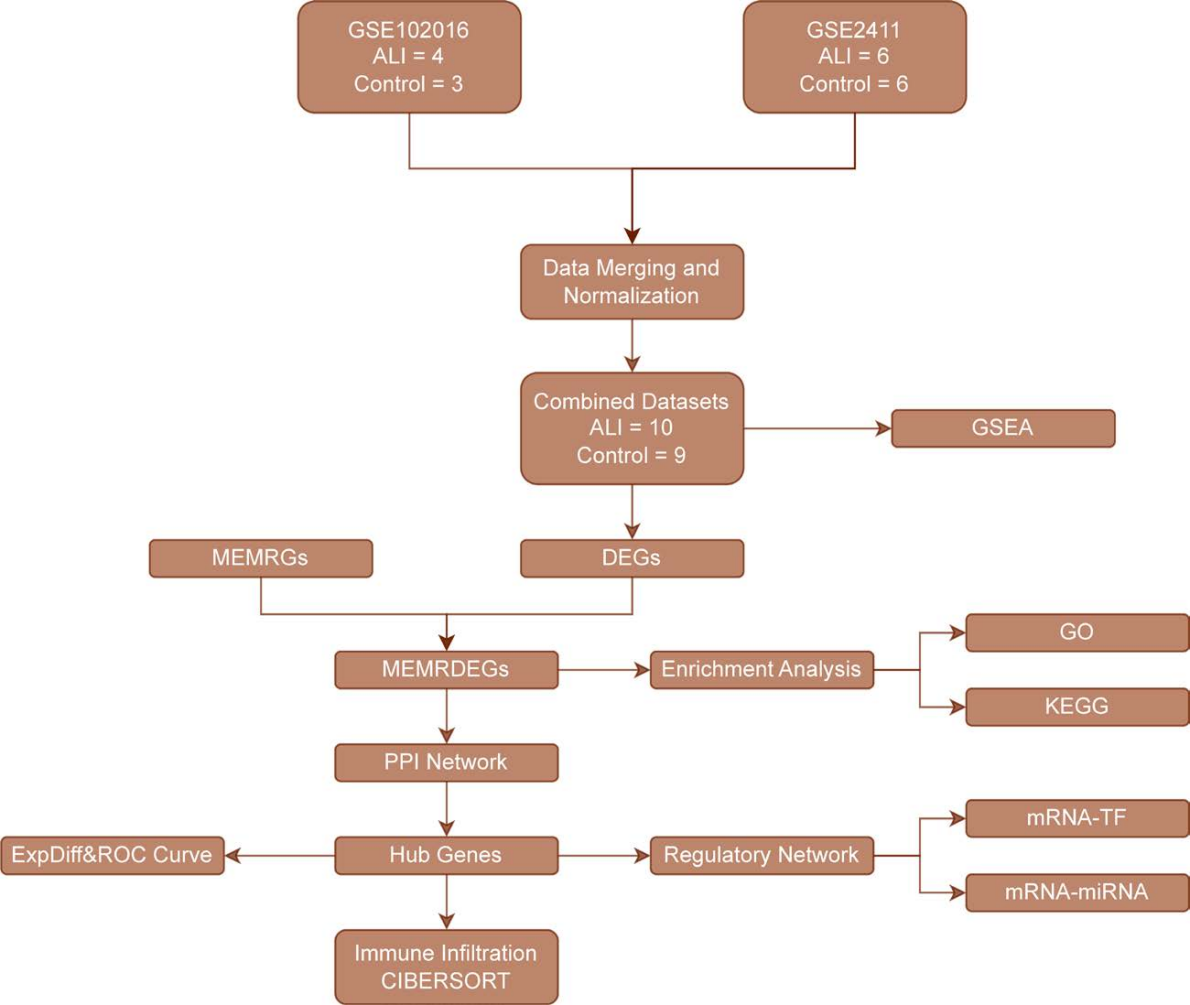
Flowchart for the comprehensive analysis of MEMRDEGs. ALI = acute lung injury, CIBERSORT = Cell-type Identification By Estimating Relative Subsets Of RNA Transcripts, DEGs = differentially expressed genes, GO = Gene Ontology, GSEA = gene set enrichment analysis, KEGG = Kyoto Encyclopedia of Genes and Genomes, MEMRDEGs = mitochondrial energy metabolism-related differentially expressed genes, MEMRGs = mitochondrial energy metabolism-related genes, mRNA-miRNA = messenger RNA–microRNA, mRNA-TF = messenger RNA–transcription factor, PPI = protein-protein interaction, ROC = receiver operating characteristic, TF = transcription factor.

To minimize batch effects between datasets, the sva package^[12]^ was applied to correct for batch effects, combining GSE102016 and GSE2411 into a combined dataset. The final combined dataset included 10 ALI samples and 9 control samples. Normalization, probe annotation, and further data processing were carried out using the limma package.^[13]^ To assess the success of batch correction and data normalization, principal component analysis (PCA)^[14]^ was implemented on the expression data pre- and post-correction. PCA reduces dimensionality by identifying principal components, enabling high-dimensional data to be transformed into a lower-dimensional format for clearer visualization in 2 or 3 dimensions. The overall analytical workflow of this study is illustrated in Figure 1.

### 2.2. Identification of MEMRDEGs in ALI

Samples were assigned to groups: ALI samples and control samples. Differential expression analysis was implemented utilizing the limma package. Genes with logFC > 0.5 and adj.*p* < .05 were defined as differentially expressed genes (DEGs). Specifically, genes with logFC > 0.5 and adj.*p* < .05 were considered upregulated, whereas those with logFC < −0.5 and adj.*p* < .05 were considered downregulated. The Benjamini–Hochberg (BH) approach was utilized to control the false discovery rate. Differential expression results were visualized using a volcano plot created with the ggplot2 package. To identify MEMRDEGs related to ALI, the DEGs identified above were intersected with the previously compiled list of MEMRGs. The overlap between DEGs and MEMRGs was visualized using a Venn diagram. A heatmap of MEMRDEG expression profiles was then constructed using the pheatmap package.

### 2.3. Functional and pathway enrichment analyses

Gene Ontology (GO) analysis^[15]^ is a commonly used approach for high-throughput functional enrichment analyses and comprises 3 principal categories: biological process (BP), cellular components, and molecular functions (MF). The Kyoto Encyclopedia of Genes and Genomes (KEGG)^[16]^ is a comprehensive database providing compiled data on genomes, molecular pathways, disease mechanisms, and pharmaceuticals. GO and KEGG analyses for the MEMRDEGs were implemented with clusterProfiler.^[17]^ *P* < .05 and false discovery rate (qvalue) < 0.25 were deemed statistically significant, with *P* value correction performed using the BH approach.

### 2.4. Gene set enrichment analysis (GSEA)

GSEA^[18]^ is a widely used computational approach for evaluating the statistical significance of predefined gene sets and the concordant differences in expression between 2 biological states. By ranking genes based on their correlation with a given phenotype, GSEA assesses whether a gene set’s members are evenly dispersed throughout the ranked list or enriched at the extremes, thereby indicating their collective involvement in the phenotypic outcome. All genes from the integrated GEO datasets were ranked based on their logFC values. GSEA was then implemented using the clusterProfiler package on the entire ranked gene list. The analysis parameters were set as follows: a random seed of 2020, 1000 permutations, and gene sets comprising between 10 and 500 genes. The curated *c2* gene sets were retrieved from MSigDB^[19]^ (https://www.gsea-msigdb.org/gsea/msigdb), specifically using the Cp.All.V2022.1.Hs.symbols collection. Significance thresholds for GSEA were defined as adj.*p* < .05 and *q* value < 0.25; both criteria had to be met for a gene set to be considered significantly enriched. The BH procedure was applied to adjust *P* values for multiple comparisons.

### 2.5. Protein-protein interaction (PPI) network construction

The PPI network represents the complex web of physical and functional interactions among proteins, which are fundamental to a wide range of BP, including gene expression regulation, cell cycle control, signal transduction, and cellular metabolism. Comprehensive analysis of protein interactions provides crucial insights into how proteins function within biological systems, how signaling and metabolic pathways are coordinated under physiological and pathological conditions, and how functional relationships among proteins can be mapped. To investigate the interactions among the identified MEMRDEGs, STRING^[20]^ (https://string-db.org/) was utilized to establish the PPI network. An interaction score threshold of >0.400 (medium confidence) was set as the minimum interaction confidence threshold to include predicted or experimentally validated interactions. This PPI network was used to identify tightly connected clusters, which may represent functional protein complexes or modules with specific biological roles. Genes that demonstrated significant interaction relationships within the network were selected for further analysis. To determine key hub genes, 5 algorithms available in the CytoHubba plug-in of Cytoscape^[21]^ were applied: degree, maximal clique centrality (MCC), maximum neighborhood component (MNC), closeness, and edge percolated component (EPC).^[22]^ The scores were computed using these algorithms, and the top 10 genes from each ranking were extracted. Data from the 5 algorithms were integrated, and a Venn diagram was constructed to identify genes that were consistently ranked as top candidates across multiple algorithms. Genes present in the intersection of these rankings were defined as key hub genes in ALI.

### 2.6. Establishment of the regulatory network

Transcription factors (TFs) modulate gene expression by interacting with hub genes at the transcriptional level. To identify potential TFs regulating the hub genes identified in this study, ChIPBase^[23]^ (http://rna.sysu.edu.cn/chipbase/) was queried. Only TFs with a combined total of more than 4 occurrences in the “Upstream sample count” and “Downstream sample count” were retained for further analysis. The interactions between these TFs and the hub genes were visualized as an messenger RNA–transcription factor (mRNA-TF) regulatory network using Cytoscape.

Beyond TFs, miRNAs are essential posttranscriptional modulators responsible for BP, including development, differentiation, and disease progression. Each miRNA can influence numerous target genes, and conversely, individual genes are often modulated by various miRNAs. To explore the regulatory association between hub genes and miRNAs, StarBase v3.0 was utilized to identify miRNAs related to the hub genes. Only miRNAs with a “clipExpNum” >5 were included. The resulting messenger RNA–microRNA (mRNA-miRNA) network was also mapped and visualized with Cytoscape.

### 2.7. Verification of differential gene expression and receiver operating characteristic (ROC) assessment

To further assess the expression differences of the identified hub genes between ALI samples and controls within the integrated GEO datasets, group comparison plots were generated according to their expression levels. To examine the diagnostic potential of these hub genes for differentiating ALI from control samples, ROC curve analysis was implemented with pROC. The area under the ROC curve (AUC) was calculated for each hub gene. Generally, AUCs vary between 0.5 and 1.0, with values of 0.5 to 0.7, 0.7 to 0.9, and >0.9 suggesting low, moderate, and high diagnostic accuracies.

### 2.8. Immune infiltration analysis

Cell-type Identification By Estimating Relative Subsets Of RNA Transcripts^[24]^ is a computational method according to linear-support vector regression that deconvolutes bulk transcriptomic data to predict the proportions and abundances of various immune cell types within a mixed cell population. In this study, Cell-type Identification By Estimating Relative Subsets Of RNA Transcripts was utilized in combination with the mouse signature gene matrix. Only samples with immune cell enrichment scores > 0 were retained, yielding an immune cell infiltration profile for the integrated datasets. Next, immune cell populations showing obvious differences in abundance between ALI and control samples were identified. The ggplot2 package was employed to generate group comparison plots to visualize these differences. To assess relationships among immune cell types, Spearman correlation coefficients were calculated, and a correlation heatmap was produced using pheatmap. Additionally, Spearman correlation analysis was conducted to examine associations between the hub genes and immune cell abundances, with the outcomes visualized with ggplot2.

### 2.9. Statistical analysis

All statistical tests were implemented using R v4.3.0. To compare continuous variables between 2 groups, normally distributed data were assessed using an independent Student’s *t* test, unless otherwise specified. Non-normally distributed data were compared using the Mann–Whitney *U* test (Wilcoxon rank-sum test). For comparisons across multiple groups, the Kruskal–Wallis test was utilized. Spearman correlation analysis was performed to determine the correlation coefficients between diverse variables. All tests were two-sided, with significance defined as *P *< .05.

## 3. Results

### 3.1. Merging of ALI datasets

**Figure 2.**
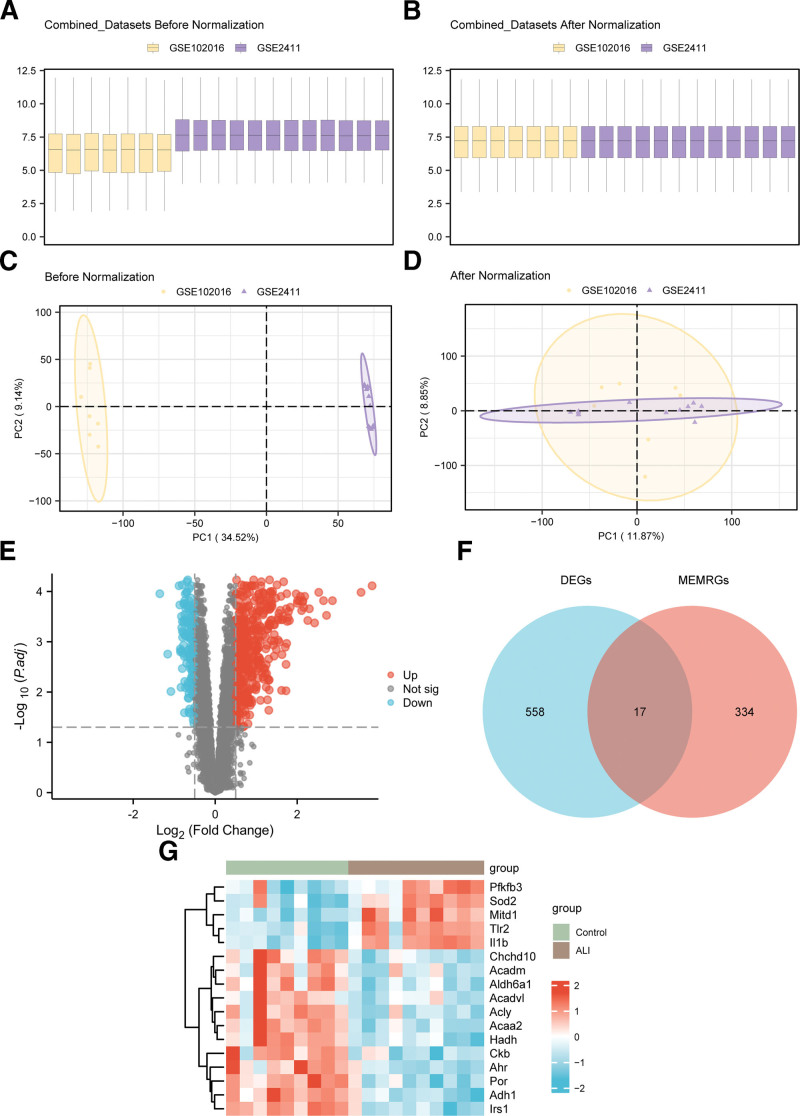
Batch effects removal of GSE102016 and GSE2411 and differential gene expression analysis. (A) Boxplots of GEO combined datasets distribution before batch removal; (B) go to the post-batch integrated GEO datasets (combined datasets) distribution boxplots; (C) 2D PCA plot of the integrated GEO datasets (combined datasets) before debatched; (D) 2D PCA plot of the integrated GEO datasets (combined datasets) after debatching. The acute lung injury (ALI) dataset GSE102016 is yellow, and the acute lung injury (ALI) dataset GSE2411 is purple; (E) volcano plot of differentially expressed genes in ALI samples and control samples in combined GEO datasets; (F) DEGs and MEMRGs Venn diagram in the integrated GEO datasets (combined datasets); (G) heat map of MEMRDEGs in the integrated GEO datasets. In the heat map group, green is the control sample, and brown is the ALI sample. In the heat map, red represents high expression and blue represents low expression. DEGs = differentially expressed genes, GEO = Gene Expression Omnibus, MEMRDEGs = mitochondrial energy metabolism-related differentially expressed genes, MEMRGs = mitochondrial energy metabolism-related genes, PCA = principal component analysis.

First, sva was utilized for batch correction between GSE102016 and GSE2411, yielding unified combined GEO datasets. To assess the effectiveness of batch correction, distribution boxplots (Fig. 2A and B) were constructed to compare gene expression pre- and post-batch correction. Secondly, PCA plots (Fig. 2C and D) were employed to visualize the distribution of low-dimensional features pre- and post-batch correction. The findings demonstrated that batch effects were successfully attenuated, yielding a more comparable and integrated dataset for subsequent analysis.

### 3.2. The association between MEMRDEGs and ALI

The combined GEO dataset was assigned to 2 groups: ALI samples and controls. Differential expression analysis between these groups was implemented with limma. Overall, 575 DEGs were identified using the thresholds logFC > 0.5 and adj.*p* < .05. Among them, 431 genes were upregulated (logFC > 0.5, adj.*p* < .05) and 144 genes were downregulated (logFC < −0.5, adj.*p* < .05). These results are visualized in a volcano plot (Fig. 2E).

To identify MEMRDEGs, the list of DEGs was intersected with the previously curated set of MEMRGs, and overlap among groups was depicted with a Venn diagram (Fig. 2F). This intersection yielded 17 MEMRDEGs, among which 5 MEMRDEGs (*Pfkfb3, Sod2, Mitd1, Tlr2,* and *Il1b*) were upregulated, while 12 MEMRDEGs (*Acaa2, Adh1, Acdm, Acly, Aldh6a1, Ahr, Por, Hadh, Acadvl, Chchd10, Irs1,* and *Ckb*) were downregulated. To further depict the expression patterns of these MEMRDEGs between ALI and controls, a heatmap was generated using the pheatmap package, as shown in Figure 2G.

### 3.3. GO and KEGG analyses

GO and KEGG were applied to examine the functional roles of the 17 identified MEMRDEGs in ALI. The enrichment analyses covered 3 GO domains: BP, CC, and MF, alongside relevant KEGG pathways. Detailed enrichment data are provided in Table 2. The analyses revealed that the MEMRDEGs were predominantly enriched in pathways and processes related to fatty acid oxidation, lipid oxidation, fatty acid metabolic process, small molecule catabolic process, and fatty acid beta-oxidation. For CC, significant enrichment was observed in the mitochondrial matrix, mitochondrial inner membrane, nucleoid, mitochondrial nucleoid, and receptor complexes. In terms of MF, key enrichments included acyl-CoA dehydrogenase activity, fatty acid derivative binding, fatty-acyl-CoA binding, flavin adenine dinucleotide binding, and acyl-CoA binding. KEGG analysis further indicated significant enrichment in pathways such as fatty acid degradation and metabolism, valine/leucine/isoleucine degradation, alcoholic liver disease, and fatty acid elongation.

**Table 2:** Result of GO and KEGG enrichment analysis for MEMRDEGs.

Ontology	ID	Description	GeneRatio	BgRatio	*P* value	*p*.adjust	qvalue
BP	GO:0019395	Fatty acid oxidation	6/17	113/28,814	3.80 e−11	2.52 e−08	9.23 e−09
BP	GO:0034440	Lipid oxidation	6/17	121/28,814	5.77 e−11	2.52 e−08	9.23 e−09
BP	GO:0006631	Fatty acid metabolic process	8/17	446/28,814	6.66 e−11	2.52 e−08	9.23 e−09
BP	GO:0044282	Small molecule catabolic process	7/17	334/28,814	4.65 e−10	1.32 e−07	4.83 e−08
BP	GO:0006635	Fatty acid beta-oxidation	5/17	76/28,814	6.74 e−10	1.53 e−07	5.61 e−08
CC	GO:0005743	Mitochondrial inner membrane	5/17	460/28,739	5.43 e−06	1.57 e−04	7.43 e−05
CC	GO:0005759	Mitochondrial matrix	4/17	288/28,739	2.12 e−05	3.08 e−04	1.45 e−04
CC	GO:0009295	Nucleoid	2/17	48/28,739	3.66 e−04	2.65 e−03	1.25 e−03
CC	GO:0042645	Mitochondrial nucleoid	2/17	48/28,739	3.66 e−04	2.65 e−03	1.25 e−03
CC	GO:0043235	Receptor complex	3/17	423/28,739	1.85 e−03	1.07 e−02	5.05 e−03
MF	GO:0003995	Acyl-CoA dehydrogenase activity	2/17	10/28,275	1.53 e−05	8.60 e−04	2.73 e−04
MF	GO:0050660	Flavin adenine dinucleotide binding	3/17	86/28,275	1.79 e−05	8.60 e−04	2.73 e−04
MF	GO:0000062	Fatty-acyl-CoA binding	2/17	26/28,275	1.10 e−04	2.63 e−03	8.35 e−04
MF	GO:1901567	Fatty acid derivative binding	2/17	27/28,275	1.18 e−04	2.63 e−03	8.35 e−04
MF	GO:0120227	Acyl-CoA binding	2/17	29/28,275	1.37 e−04	2.63 e−03	8.35 e−04
KEGG	mmu00071	Fatty acid degradation	5/15	52/9000	1.52 e−08	1.46 e−06	1.15 e−06
KEGG	mmu00280	Valine, leucine, and isoleucine degradation	4/15	57/9000	1.87 e−06	8.43 e−05	6.65 e−05
KEGG	mmu01212	Fatty acid metabolism	4/15	62/9000	2.63 e−06	8.43 e−05	6.65 e−05
KEGG	mmu04936	Alcoholic liver disease	4/15	141/9000	6.89 e−05	1.65 e−03	1.31 e−03
KEGG	mmu00062	Fatty acid elongation	2/15	29/9000	1.03 e−03	1.97 e−02	1.55 e−02

BP = biological process, CC = cellular component, GO = Gene Ontology, KEGG = Kyoto Encyclopedia of Genes and Genomes, MEMRDEGs = mitochondrial energy metabolism-related differentially expressed genes, MF = molecular function.

**Figure 3.**
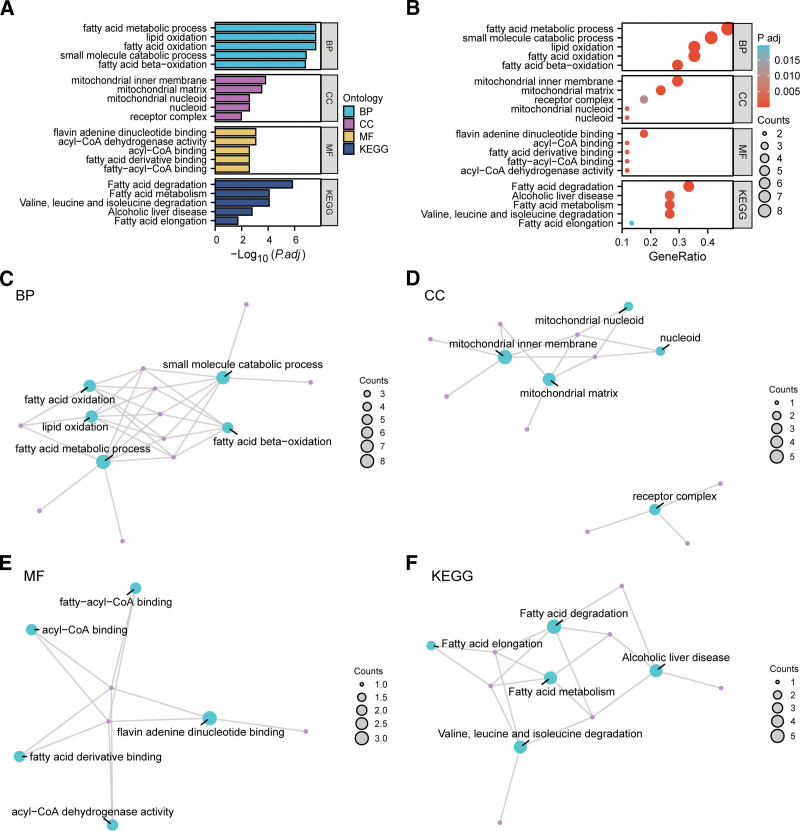
GO and KEGG enrichment analysis for MEMRDEGs. (A, B) GO and pathway (KEGG) enrichment analysis results of MEMRDEGs bar graph (A) and bubble plot (B) show: BP, CC, MF, and biological pathway (KEGG). GO terms and KEGG terms are shown on the ordinate; (C–F) GO and pathway (KEGG) enrichment analysis results of MEMRDEGs: BP (C), CC (D), MF (E), and KEGG (F). Blue nodes represent items, purple nodes represent molecules, and the lines represent the relationship between items and molecules. The bubble size in the bubble plot represents the number of genes, and the color of the bubble represents the size of the adj. *P* value, the redder the color, the smaller the adj. *P* value, and the bluer the color, the larger the adj. *P* value. The screening criteria for Gene Ontology (GO) and pathway (KEGG) enrichment analysis were adj.*p* < .05 and FDR value (*q* value) < 0.25 were considered statistically significant, and the *P* value correction method was Benjamini–Hochberg (BH). BP = biological process, CC = cellular component, FDR = false discovery rate, KEGG = Kyoto Encyclopedia of Genes and Genomes, MEMRDEGs = mitochondrial energy metabolism-related differentially expressed genes, MF = molecular function.

GO and KEGG outcomes were depicted using bar plots (Fig. 3A) and bubble plots (Fig. 3B). Network diagrams displaying the relationships among BP, CC, MF, and KEGG pathways were generated (Fig. 3C–F). In these diagrams, edges represent gene-term associations, and node size represents the number of genes in the respective term. Collectively, these findings indicate a predominant gene enrichment in the fatty acid metabolic process within the BP category.

### 3.4. GSEA for ALI

**Figure 4.**
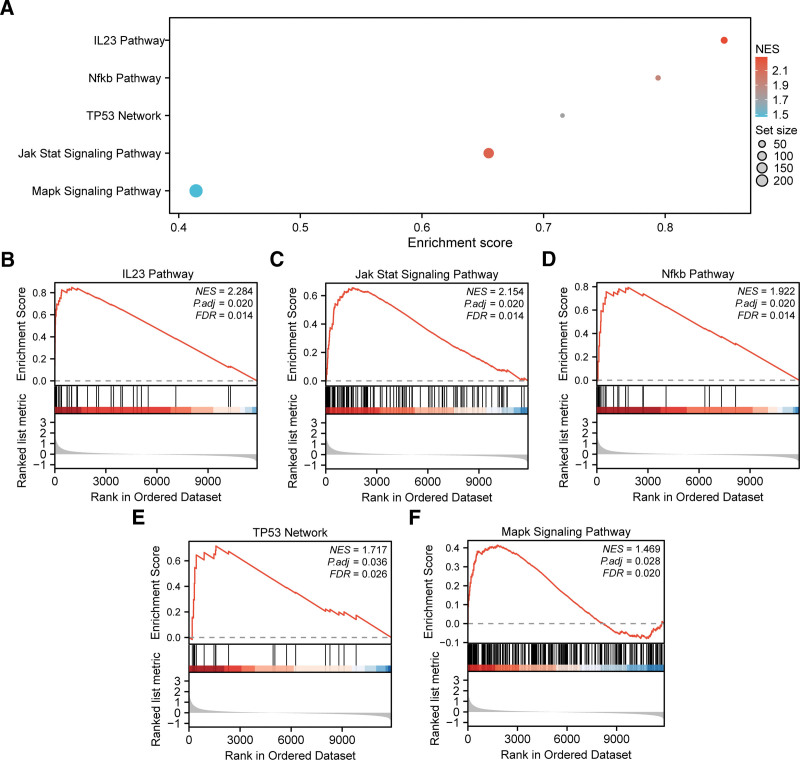
GSEA for combined datasets. (A) GSEA 5 biological functions bubble plot presentation of the integrated GEO datasets; (B–F) GSEA showed that all genes were significantly enriched in IL23 pathway (B), Jak Stat signaling pathway (C), Nfkb pathway (D), TP53 network (E), and Mapk signaling pathway (F). In the bubble plot, the size of the bubble represents the number of enriched genes, and the color of the bubble represents the size of the Normalized Enrichment Score (NES) value. The more red the color is, the greater the NES value is, and the more blue the color is, the smaller the NES value is. The screening criteria of GSEA were adj.*p* < .05 and FDR value (*q* value) < 0.25 were considered statistically significant, and the *P* value correction method was Benjamini–Hochberg (BH). FDR = false discovery rate, GEO = Gene Expression Omnibus, GSEA = gene set enrichment analysis.

To comprehensively evaluate the functional influence of global expression patterns in the combined GEO dataset on ALI, GSEA was performed. This method assessed the distribution of all genes according to their expression profiles and their roles in key BP, CC, and MF (Fig. 4A, Table 3). The GSEA results indicated a predominant gene enrichment in several critical signaling pathways and BP associated with ALI pathogenesis, including the IL23 pathway (Fig. 4B), Jak Stat pathway (Fig. 4C), Nfkb pathway (Fig. 4D), TP53 network (Fig. 4E), and Mapk pathway (Fig. 4F).

**Table 3:** Results of GSEA for combined datasets.

ID	Set size	Enrichment score	NES	*P* value	*p*.adjust	*q* value
WP_OVERVIEW_OF_PROINFLAMMATORY_AND_PROFIBROTIC_MEDIATORS	84	8.57 e−01	2.69 e+00	1.42 e−03	1.97 e−02	1.42 e−02
WP_NETWORK_MAP_OF_SARSCOV2_SIGNALING_PATHWAY	185	7.56 e−01	2.62 e+00	1.25 e−03	1.97 e−02	1.42 e−02
REACTOME_SIGNALING_BY_INTERLEUKINS	393	6.93 e−01	2.58 e+00	1.10 e−03	1.97 e−02	1.42 e−02
REACTOME_INTERFERON_SIGNALING	151	7.53 e−01	2.55 e+00	1.29 e−03	1.97 e−02	1.42 e−02
REACTOME_INTERLEUKIN_10_SIGNALING	42	9.07 e−01	2.54 e+00	1.57 e−03	1.97 e−02	1.42 e−02
WP_SARSCOV2_INNATE_IMMUNITY_EVASION_AND_CELLSPECIFIC_IMMUNE_RESPONSE	60	8.49 e−01	2.52 e+00	1.48 e−03	1.97 e−02	1.42 e−02
REACTOME_INTERFERON_ALPHA_BETA_SIGNALING	55	8.62 e−01	2.52 e+00	1.49 e−03	1.97 e−02	1.42 e−02
REACTOME_CHEMOKINE_RECEPTORS_BIND_CHEMOKINES	47	8.77 e−01	2.49 e+00	1.52 e−03	1.97 e−02	1.42 e−02
KEGG_CYTOKINE_CYTOKINE_RECEPTOR_INTERACTION	208	7.08 e−01	2.48 e+00	1.24 e−03	1.97 e−02	1.42 e−02
REACTOME_PEPTIDE_LIGAND_BINDING_RECEPTORS	154	7.26 e−01	2.46 e+00	1.29 e−03	1.97 e−02	1.42 e−02
REACTOME_INTERLEUKIN_4_AND_INTERLEUKIN_13_SIGNALING	105	7.64 e−01	2.45 e+00	1.38 e−03	1.97 e−02	1.42 e−02
KEGG_TOLL_LIKE_RECEPTOR_SIGNALING_PATHWAY	88	7.64 e−01	2.41 e+00	1.40 e−03	1.97 e−02	1.42 e−02
WP_TOLLLIKE_RECEPTOR_SIGNALING_PATHWAY	89	7.65 e−01	2.41 e+00	1.41 e−03	1.97 e−02	1.42 e−02
REACTOME_INTERFERON_GAMMA_SIGNALING	72	7.91 e−01	2.40 e+00	1.47 e−03	1.97 e−02	1.42 e−02
WP_TYPE_II_INTERFERON_SIGNALING	37	8.70 e−01	2.38 e+00	1.61 e−03	1.97 e−02	1.42 e−02
PID_IL23_PATHWAY	35	8.48 e−01	2.28 e+00	1.63 e−03	1.97 e−02	1.42 e−02
KEGG_JAK_STAT_SIGNALING_PATHWAY	120	6.55 e−01	2.15 e+00	1.33 e−03	1.97 e−02	1.42 e−02
BIOCARTA_NFKB_PATHWAY	21	7.94 e−01	1.92 e+00	1.78 e−03	1.97 e−02	1.42 e−02
WP_TP53_NETWORK	19	7.16 e−01	1.72 e+00	6.98 e−03	3.62 e−02	2.61 e−02
KEGG_MAPK_SIGNALING_PATHWAY	239	4.14 e−01	1.47 e+00	4.91 e−03	2.82 e−02	2.03 e−02

GSEA = gene set enrichment analysis, NES = Normalized Enrichment Score.

### 3.5. Construction of PPI network

To investigate the functional relationships among the 17 MEMRDEGs, a PPI network was established via STRING (Fig. S1A, Supplemental Digital Content, https://links.lww.com/MD/R781). The analysis revealed that 12 of the 17 MEMRDEGs formed interaction networks, specifically *Acaa2, Adh1, Sod2, Hadh, Acly, Aldh6a1, Acadvl, Acadm, Il1b, Pfkfb3, Irs1,* and *Tlr2.* To identify key hub genes, CytoHubba was used to calculate network centrality scores using 5 distinct algorithms: MNC, degree, MCC, closeness, and EPC. The top 10 MEMRDEGs ranked by each algorithm were visualized to illustrate their network importance: MCC (Fig. S1B, Supplemental Digital Content, https://links.lww.com/MD/R781), degree (Fig. S1C, Supplemental Digital Content, https://links.lww.com/MD/R781), MNC (Fig. S1D, Supplemental Digital Content, https://links.lww.com/MD/R781), EPC (Fig. S1E, Supplemental Digital Content, https://links.lww.com/MD/R781), and closeness (Fig. S1F, Supplemental Digital Content, https://links.lww.com/MD/R781). In these visualizations, node color gradients from yellow to red indicate ranking from low to high. Finally, the overlapping genes identified by all 5 algorithms were determined and visualized using a Venn diagram (Fig. S1G, Supplemental Digital Content, https://links.lww.com/MD/R781). This intersection yielded 9 hub genes, including *Acaa2, Acadm, Acadvl, Acly, Hadh, Sod2, Aldh6a1, Irs1,* and *Adh1.*

### 3.6. Establishment of regulatory networks

Firstly, TFs predicted to bind to the identified hub genes were derived from ChIPBase. In accordance with these data, the mRNA-TF network was established and visualized with Cytoscape (Fig. S2A, Supplemental Digital Content, https://links.lww.com/MD/R781). Nine hub genes and 31 TFs were incorporated into this network, as detailed in Table S2, Supplemental Digital Content, https://links.lww.com/MD/R782. In addition, miRNAs potentially regulating the hub genes were identified through TarBase. The mRNA-miRNA network was then established and visualized in Cytoscape (Fig. S2B, Supplemental Digital Content, https://links.lww.com/MD/R781). Four hub genes and 43 miRNAs were included in this network, as summarized in Table S3, Supplemental Digital Content, https://links.lww.com/MD/R782.

### 3.7. Verification of differential gene expression and ROC assessment

**Figure 5.**
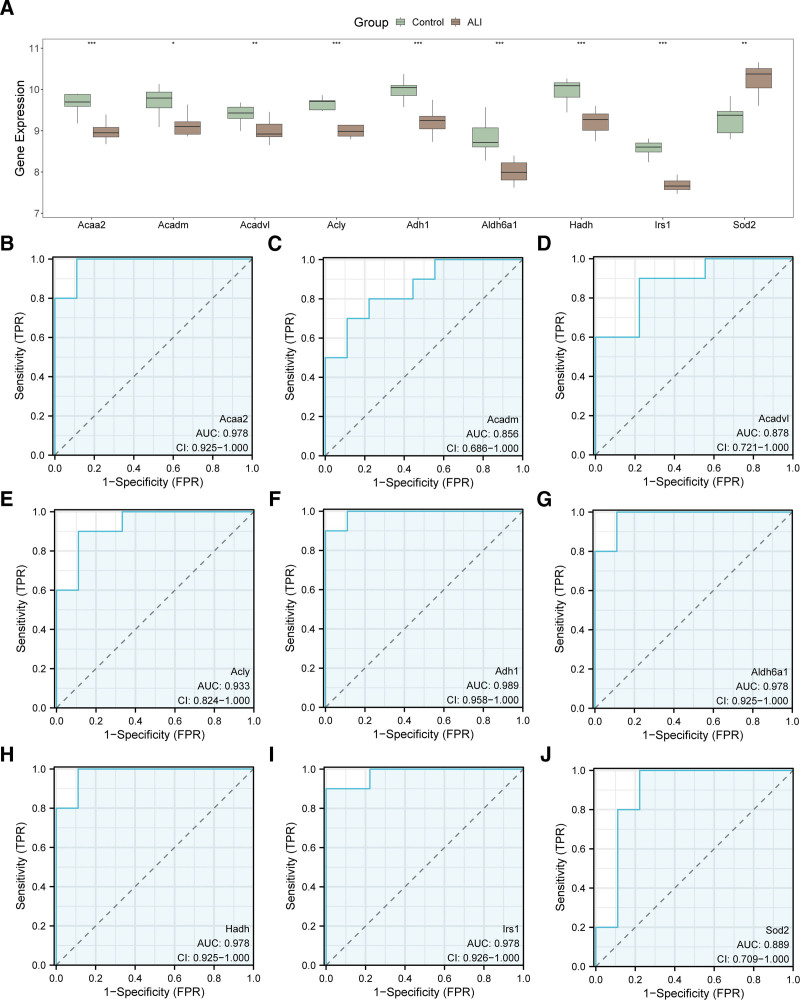
Differential expression validation and ROC curve analysis. (A) Group comparison plots of hub genes in ALI samples and control samples from the combined GEO datasets; (B–J) hub genes *Acaa2* (B), *Acadm* (C), *Acadvl* (D), *Acly* (E), *Adh1* (F), *Aldh6a1* (G), *Hadh* (H), ROC curves of *Irs1* (I) and *Sod2* (J) in combined GEO datasets. * stands for *P* value < .05 and is statistically significant; ** represents *P* value < .01, highly statistically significant; *** represents *P* value < .001 and highly statistically significant. In the group comparison graph, green is the control sample, and brown is the ALI sample. AUC between 0.7 and 0.9 had a certain accuracy; AUC above 0.9 had a high accuracy. ALI = acute lung injury, AUC = area under the curve, FPR = false positive rate, GEO = Gene Expression Omnibus, ROC = receiver operating characteristic, TPR = true positive rate.

To validate the differential expression of the hub genes in ALI, we compared their expression levels between ALI samples and controls within the combined GEO dataset. As illustrated in Figure 5A, all 9 hub genes demonstrated significant expression difference (*P* < .05). Specifically, *Acadm* showed notable expression difference (*P* < .05), *Acadvl* and *Sod2* showed highly significant differences (*P* < .01), and *Acaa2*, *Acly*, *Adh1*, *Aldh6a1*, *Hadh*, and *Irs1* displayed very strong significance (*P* < .001). To further evaluate their diagnostic potential, ROC curves were generated for each hub gene based on their expression levels in the combined dataset (Fig. 5B–J). The findings demonstrated that 6 hub genes (*Acaa2, Acly, Adh1, Aldh6a1, Hadh,* and *Irs1*) achieved AUC values >0.9, demonstrating strong diagnostic performance in differentiating ALI samples from controls. The remaining 3 genes (*Acadm, Acadvl,* and *Sod2*) showed moderate diagnostic performance, with AUCs of 0.7 to 0.9.

### 3.8. Immune infiltration analysis in ALI

**Figure 6.**
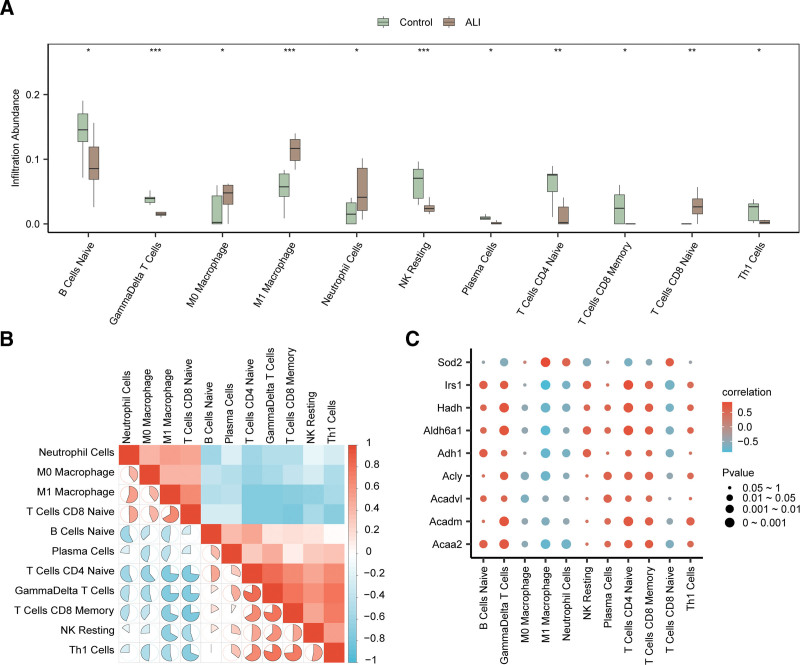
Combined datasets immune infiltration analysis by the CIBERSORT algorithm. (A) Group comparison plot of immune cells in the integrated GEO datasets (combined datasets); (B) correlation heatmap of immune cell infiltration abundance in the integrated GEO datasets (combined datasets); (C) bubble plot of the correlation between hub genes and immune cell infiltration abundance in the integrated GEO datasets (combined datasets). * represents *P* value < .05, statistically significant; ** represents *P* value < .01, highly statistically significant; *** represents *P* value < .001 and highly statistically significant. The absolute value of correlation coefficient (*r* value) below 0.3 was weak or no correlation, between 0.3 and 0.5 was weak correlation, and between 0.5 and 0.8 was moderate correlation. In the group comparison graph, green is the control sample, and brown is the ALI sample. Blue is the negative correlation, red is the positive correlation, and the depth of color represents the strength of correlation. ALI = acute lung injury, CIBERSORT = Cell-type Identification By Estimating Relative Subsets Of RNA Transcripts, GEO = Gene Expression Omnibus.

A group comparison plot was used to illustrate variations in immune cell infiltration between ALI and control samples (Fig. 6A). The analysis showed that infiltration levels of 11 immune cell types differed significantly between ALI samples and controls (*P* < .05). Specifically, 6 cell types, including naive B cells, M0 macrophages, neutrophils, plasma cells, cluster of differentiation (CD) 8 memory T cells, and Th1 cells, showed significant differences (*P* < .05). Moreover, naive CD4 and CD8 T cell displayed statistically robust differences (*P* < .01), while γδ T cells, M1 macrophages, and resting NK cells demonstrated the most significant differences (*P* < .001).

To further explore the relationships among these immune cell populations, a correlation heatmap was constructed to visualize pairwise correlations in infiltration abundance (Fig. 6B). The strongest positive correlation was observed between Th1 cell and γδ T cell (*r* = 0.79), whereas the strongest negative correlation was found between CD8 memory T cell and CD8 naive T cell (*r* = −0.77). Furthermore, the associations between the hub genes and the abundances of these immune cell types were evaluated using Spearman correlation analysis. The results were visualized with correlation bubble plots (Fig. 6C). The plots indicated that naive B cell, γδ T cell, plasma cell, CD4 naive T cell, CD8 memory T cell, and Th1 cell were positively correlated with most hub genes (*r* > 0, *P* < .05). In contrast, M0 macrophages, M1 macrophages, neutrophils, and CD8 naive T cells demonstrated significant negative correlations with the majority of hub genes (*r* < 0, *P* < .05).

## 4. Discussion

ALI is a serious clinical syndrome defined by sudden-onset respiratory failure resulting from various direct or indirect insults to the lungs, including pneumonia, sepsis, and trauma. ALI is related to high mortality and morbidity, placing a heavy burden on healthcare infrastructures globally. Epidemiological data estimate the incidence of ALI to range from 20 to 50 cases per 100,000 person-years, with mortality rates comparable to those of other life-threatening conditions such as HIV and breast cancer.^[25]^ Despite advances in understanding its complex pathophysiology, there is still no effective targeted pharmacotherapy for ALI, emphasizing the critical need for ongoing research to unravel the disease mechanisms and develop innovative treatment strategies.^[1]^

Mitochondrial dysfunction reduces adenosine triphosphate production and increases reactive oxygen species, activating nuclear factor-kappa B signaling and driving pro-inflammatory cytokine release. This, in turn, recruits macrophages and CD8 T cells, perpetuating epithelial and endothelial injury, and ultimately exacerbating ALI. This study focuses on mitochondrial energy metabolism as an essential driver of ALI development. By applying bioinformatics analyses to integrate large-scale datasets, we identified MEMRDEGs associated with ALI. The results demonstrate that these genes are significantly enriched in metabolic pathways, offering valuable insights into potential therapeutic targets for ALI. Furthermore, our findings emphasize the critical role of mitochondrial dysfunction in driving the inflammatory responses and tissue damage characteristic of ALI, suggesting that metabolic modulation could represent a promising therapeutic avenue.^[3,26]^

Our analysis revealed a total of 575 DEGs associated with ALI, of which a substantial proportion were linked to mitochondrial energy metabolism. Among these, 431 and 144 genes were upregulated and downregulated, respectively, indicating extensive and complex alterations in metabolic gene expression that may contribute to ALI pathogenesis.^[27]^ Identifying these genes is crucial, as it enhances our understanding of the BP underlying ALI and provides a foundation for the design of novel biomarkers and targeted therapies aimed at restoring mitochondrial function and improving patient outcomes.^[25]^

Pathway enrichment analyses revealed that MEMRDEGs were primarily enriched in fatty acid oxidation and lipid metabolism. Enhanced fatty acid oxidation may promote excessive reactive oxygen species production, thereby aggravating alveolar injury and contributing to the progression of ALI. These observations emphasize the roles of mitochondrial energy homeostasis in the pathogenesis of ALI and suggest that targeting metabolic pathways may offer promising therapeutic opportunities.^[28]^ Moreover, the relationship between mitochondrial dysfunction and inflammation in lung injury is particularly significant, as inflammation can further disrupt metabolic processes, leading to a vicious cycle that exacerbates tissue damage and worsens clinical outcomes.^[29]^ This highlights the potential of metabolic modulators as innovative treatments that could simultaneously address both the metabolic and inflammatory aspects of ALI.^[30]^

In addition, our analysis of the immune landscape in ALI demonstrated significant changes in the infiltration levels of various immune cell types, including M0 macrophages and CD8 T cells. An increase in M0 macrophages may indicate impaired differentiation into pro-resolving M2 phenotypes, thereby prolonging inflammation. Elevated CD8 T cells could further exacerbate tissue injury through cytotoxic activity. These observations emphasize the complex crosstalk between mitochondrial dysfunction and immune cell regulation in the pathophysiology of ALI.^[3]^ The altered immune microenvironment may contribute to the progression of ALI and presents opportunities for immunomodulatory interventions aimed at improving patient outcomes.^[26]^ Future studies should further investigate the mechanistic interactions between these immune cells and key DEGs to clarify their roles in ALI pathogenesis and to identify novel molecular targets for therapeutic development.^[31]^

Our study also constructed a PPI network, which identified 12 MEMRDEGs with significant interactions, ultimately leading to the recognition of 9 hub genes. These hub genes could represent focal points for future investigations, as they are likely to play key roles within the complex molecular pathways involved in ALI.^[32]^ Targeting these hub genes could facilitate the development of innovative therapeutic strategies that address not only mitochondrial dysfunction but also modulate dysregulated immune responses in ALI.^[33]^ In addition, the identification of regulatory networks, including mRNA-miRNA and mRNA-TF interactions, provides deeper insights into the regulatory landscape underlying ALI pathogenesis. This information lays a foundation for precision medicine approaches that could tailor treatments based on individual genetic and molecular profiles.^[34]^

This study identified pivotal genes related to mitochondrial energy metabolism in a mouse model of ALI. Corresponding homologous genes exist in humans, exhibiting functional conservation in energy metabolism regulation and inflammation-related pathways. This provides a reference foundation for translating molecular alterations observed in animal models to the understanding of human ALI. It should be noted that the findings primarily reflect molecular feature alterations at the association level, and their potential biological significance requires further validation in more targeted experimental systems. Future studies should integrate functional experiments at the cellular or animal level to explore the regulatory roles of key genes in metabolic homeostasis and inflammatory responses under inflammatory stimuli, thereby comprehensively elucidating their functions in the pathogenesis and progression of ALI.

In summary, our findings emphasize the pivotal roles of mitochondrial energy metabolism in ALI and highlight the potential of combined metabolic and immunotherapeutic interventions to improve outcomes for patients affected by this devastating condition. Continued investigation into the interplay between mitochondrial function, immune responses, and gene regulatory networks will be essential for advancing effective treatments for ALI.^[35]^

However, this study has several important limitations. Most notably, the absence of experimental validation restricts the strength of our conclusions. Although we identified significant DEGs and corresponding pathways, the reliance on mouse data and modest sample size may limit the extent to which these findings can be generalized to wider patient populations. Moreover, integrating multiple datasets carries the inherent risk of residual batch effects, which could introduce variability and influence the reliability of the findings. These constraints emphasize the importance of further experimental studies and larger, well-designed cohorts to validate and extend our observations and confirm their clinical applicability.

## 5. Conclusion

Taken together, our study enhances knowledge of the molecular pathways involved in ALI, revealing key DEGs, enriched pathways, and immune cell alterations that advance our understanding of the disease. These findings may support the discovery of new biomarkers and therapeutic targets. Future research should focus on experimental validation of these findings and further investigation of their relevance within the framework of precision medicine to enhance ALI management.

## Author contributions

**Conceptualization:** Yao Yang.

**Methodology:** Yao Yang.

**Data curation:** Yao Yang.

**Formal analysis:** Yao Yang.

**Investigation:** Yao Yang.

**Software:** Yao Yang.

**Validation:** Yao Yang.

**Visualization:** Yao Yang.

**Writing – original draft:** Yao Yang.

**Writing – review & editing:** Yao Yang.

## Supplementary Material
