# Supplementary material for: Analysis of differentially expressed genes related to acute lung injury and their role in metabolic pathways: An integrative study using GEO data
Source: Medicine (Baltimore). 2026 May 1;105(18):e48519. doi: 10.1097/MD.0000000000048519 (PMC13138442; doi:10.1097/MD.0000000000048519)
Supplement: Supplementary file 1 [file medi-105-e48519-s001.pdf]

Table S1. List of 351 Mouse MEMRGs Mapped from Human MEMRGs

Acadl
Aldh18a1
Ppat
Gapdh
Gm20899
Aldh2
Acsl4
Pparg
Cyp2u1
Pfkp
Acsl1
Gpi1
Ndufs6
Gm6415
Pcx
Mdh2
Cyc1
Pfkfb2
Acat1
Cpt1b
Acaa2
Ndufb4
Gm3244
Gm3873
Acaa1a
Mdh1b
Ndufs8
Ndufab1
LOC102634451
Aldh3b1
Cox5a
Atp4a
Ogdh
Acsbg1
LOC102640946
Ndufb9
Ndufs2
Aldh1b1
Aldh1a2
Ndufv1
Sdha
Ppargc1b

Ndufa9
Cyp4a31
Cyp4a32
Cyp4a10
Ndufb11
Ndufa12
Pfkfb4
Idh1
Acadsb
Atp4b
Aldh3a1
Echs1
Acads
Ppan
Sdhc
Mdh1
Pgm1
Pfkfb3
Ndufb5
Aldh1l2
Ndufs3
Pklr
Ndufa8
Hadha
Pdhx
Ndufb3
Aldh1l1
Cpt1a
Cyp4a12a
Cyp4a12b
Ppargc1a
Adh1
Ehhadh
Ndufb10
Oxct1
Acadm
Aldh4a1
Cs
Pdhb
Ndufa7
Ndufa5
Cox7b
Cpt1c

Uqcrfs1
Acly
Gcdh
Pfkl
Idh3g
Acox2
Ndufa10
Pfkfb1
Gapdhs
Eci1
Cox10
Aldh8a1
Aldh6a1
Ndufa11
Gm4943
Aldh9a1
Adh4
Ndufa6
Minpp1
Akr1a1
Aco1
Ndufv2
Ndufa13
Ndufa1
Dlat
Pdc
Adpgk
Idh3b
Atp12a
Eci2
Cpt2
Ahr
Hmgcl
Por
Acox1
Gck
Dlst
Cox17
Hadhb
Dld
Acox3
Sdhb
Ndufs1

Aldh3a2
Lhpp
Ppara
Acsl6
Ppard
Acsbg2
Ndufb7
Aldh5a1
Hadh
Ndufs7
Aldh1a1
Ppa2
Ndufa4
Cox15
Pfkm
Ndufc2
Bpgm
Ndufs4
Ndufv3
Acsl3
Acat3
Acat2
Aldh16a1
Sdhd
Ndufb2
Acadvl
Ndufb6
Ndufc1
Galm
Aldh7a1
Aldh1a3
Acsl5
Ndufb8
Aco2
Cox11
Ndufa2
Idh3a
Ndufa3
LOC102631912
Ppa1
C1qbp
Gfm2
Htt

Tango2
Lias
Nfatc4
Slc25a47
Etfa
Ak2
Cycs
Slc25a20
Etfb
Slc25a3
Fbxl4
Pten
Pdp1
Ucp2
Coq9
Polg
Trp53
Sod1
Tlr2
Cnr1
Sco2
Ucp1
Ucp3
Ak3
Tcf19
Dnajc19
Dnajc19-ps
Prkaca
Eif4e
Rps6kb1
Kng1
Kng2
Prkacb
Prkcz
Eif4ebp1
Rps6kb2
Klk4
Acad9
Cd200
Oma1
Ryr1
Sucla2
Tufm

Gfm1
Hibch
Tsfm
Tmem70
Opa3
Serac1
Mtif2
Cenpo
Mtif3
Lix1
1700019D03Rik
Nfu1
Lipt2
Fh1
Lipt1
Iba57
Src
Sirt3
Tpk1
Agk
Aifm1
Bdnf
Nr1h4
Crat
Ngly1
Dhtkd1
Chchd10
Gpbar1
Pkhd1
Uchl1
Gsk3b
Ass1
Mfn2
Atp7b
Irs1
Pah
Scn9a
Sod2
Tpi1
Btd
Kcnk9
Vdac1
Ocrl

Mipep
Krit1
Rars2
Sumf1
Mfsd7c
Slc27a1
Bola3
Szt2
Mitd1
Fam210b
Esr1
Psen1
Atp2a2
Cat
Cbs
Foxo1
Notch3
Adk
Apoe
Eif2ak3
Gls
Pgk1
Stim1
Casp7
Hprt
Hspd1
Kcnj2
Pkd2
Slc25a4
Ywhae
Adora2b
Hspa9
Htr2a
Il1b
Ins2
Kcnj5
Vcl
Fxn
Hk2
Neu1
Prdx6
Ryr2
Xbp1

Ywhaz
Adora2a
Htr3a
Huwe1
Bsg
Ckb
Cox4i1
Eif2s1
Igf2bp2
Shmt1
Tfam
Uqcrc2
Atg7
Bad
Clock
Gdap1
Pnpla6
Dbt
Epo
Esrrg
Htr2b
Id2
Lrpprc
Nme4
Pdk4
Soat2
Uqcrc1
Bicd2
Inf2
Ndufaf1
Prok2
Ryr3
Slc25a5
Surf1
Glrx5
Immt
Mavs
Ssbp1
Dmrt1
Ndufaf3
Sirt5
Cox7a2l
Ola1

Smpd2
Timmdc1
Sirt4
Slc25a27
Acad10
Mrs2
Fahd1
Ociad1

Table S2. Predicted Transcription Factor–Hub Gene Regulatory Network Derived from ChIPBase.

mRNA	TF
Acaa2	ARNTL
Acaa2	KLF4
Acaa2	MYC
Acaa2	PPARA
Acaa2	PPARG
Acadm	CTCF
Acadm	ESRRB
Acadm	EZH2
Acadm	HDAC3
Acadm	KLF4
Acadm	PPARG
Acadm	RNF2
Acadm	SP1
Acadm	SPI1
Acadm	SUZ12
Acadvl	CTCF
Acadvl	HDAC3
Acadvl	JUND
Acadvl	KLF4
Acadvl	PCGF6
Acadvl	RAD21
Acadvl	RXRA-B
Acadvl	SMC1A
Acadvl	SPI1
Acly	ARNTL
Acly	CTCF
Acly	ESRRB
Acly	FLI1
Acly	HDAC3
Acly	KLF4
Acly	MYC
Acly	PCGF6
Acly	POU5F1
Acly	RAD21
Acly	SMC1A
Acly	SP1
Acly	SPI1
Acly	STAT5
Acly	STAT5A
Adh1	ARNTL
Adh1	HDAC3

Aldh6a1	RAD21
Aldh6a1	TBP
Aldh6a1	ARNTL
Aldh6a1	BHLHE40
Aldh6a1	CTCF
Aldh6a1	EBF1
Hadh	CEBPB
Hadh	CTCF
Hadh	ESR1
Hadh	HDAC3
Hadh	KLF4
Hadh	PPARG
Hadh	RAD21
Hadh	SMC1A
Hadh	SPI1
Irs1	BRD4
Irs1	CEBPA
Irs1	CEBPB
Irs1	KLF4
Irs1	MYOD1
Irs1	POLR2A
Irs1	RNF2
Irs1	SPI1
Irs1	SUZ12
Sod2	ESR1
Sod2	MYC
Sod2	SP1

Table S3. Predicted miRNA–Hub Gene Regulatory Relationships from TarBase.

mRNA	miRNA
Acadm	mmu-miR-15b-5p
Acadm	mmu-miR-195a-5p
Acadm	mmu-miR-291a-5p
Acadm	mmu-miR-19b-3p
Acadm	mmu-miR-15a-5p
Acadm	mmu-miR-16-5p
Acadm	mmu-miR-322-5p
Acadm	mmu-miR-19a-3p
Acadm	mmu-miR-410-3p
Acadm	mmu-miR-291b-5p
Acadm	mmu-miR-497a-5p
Acadm	mmu-miR-495-3p
Acadm	mmu-miR-374b-5p
Acadm	mmu-miR-340-5p
Acadm	mmu-miR-1192
Acadm	mmu-miR-6240
Acly	mmu-miR-27b-3p
Acly	mmu-miR-135a-5p
Acly	mmu-miR-141-3p
Acly	mmu-miR-129-5p
Acly	mmu-miR-19b-3p
Acly	mmu-miR-200a-3p
Acly	mmu-miR-27a-3p
Acly	mmu-miR-135b-5p
Acly	mmu-miR-19a-3p
Acly	mmu-miR-381-3p
Acly	mmu-miR-450a-5p
Acly	mmu-miR-673-5p
Acly	mmu-miR-1955-5p
Acly	mmu-miR-208b-5p
Irs1	mmu-miR-29b-3p
Irs1	mmu-miR-29b-3p
Irs1	mmu-miR-29a-3p
Irs1	mmu-miR-29a-3p
Irs1	mmu-miR-29c-3p
Irs1	mmu-miR-29c-3p
Irs1	mmu-miR-7a-5p
Irs1	mmu-miR-7b-5p
Irs1	mmu-miR-487b-3p
Irs1	mmu-miR-495-3p
Irs1	mmu-miR-758-3p
Irs1	mmu-miR-770-5p

Irs1	mmu-miR-1192
Irs1	mmu-miR-3063-3p
Irs1	mmu-miR-3102-3p
Irs1	mmu-miR-3113-5p
Irs1	mmu-miR-493-5p
Irs1	mmu-miR-219a-2-3p
Sod2	mmu-miR-669d-5p
Sod2	mmu-miR-3070-3p
