# Supplementary material for: Analysis of differentially expressed genes related to acute lung injury and their role in metabolic pathways: An integrative study using GEO data
Source: Medicine (Baltimore). 2026 May 1;105(18):e48519. doi: 10.1097/MD.0000000000048519 (PMC13138442; doi:10.1097/MD.0000000000048519)
Supplement: Supplementary file 2 [file medi-105-e48519-s002.pdf]

Supplementary data

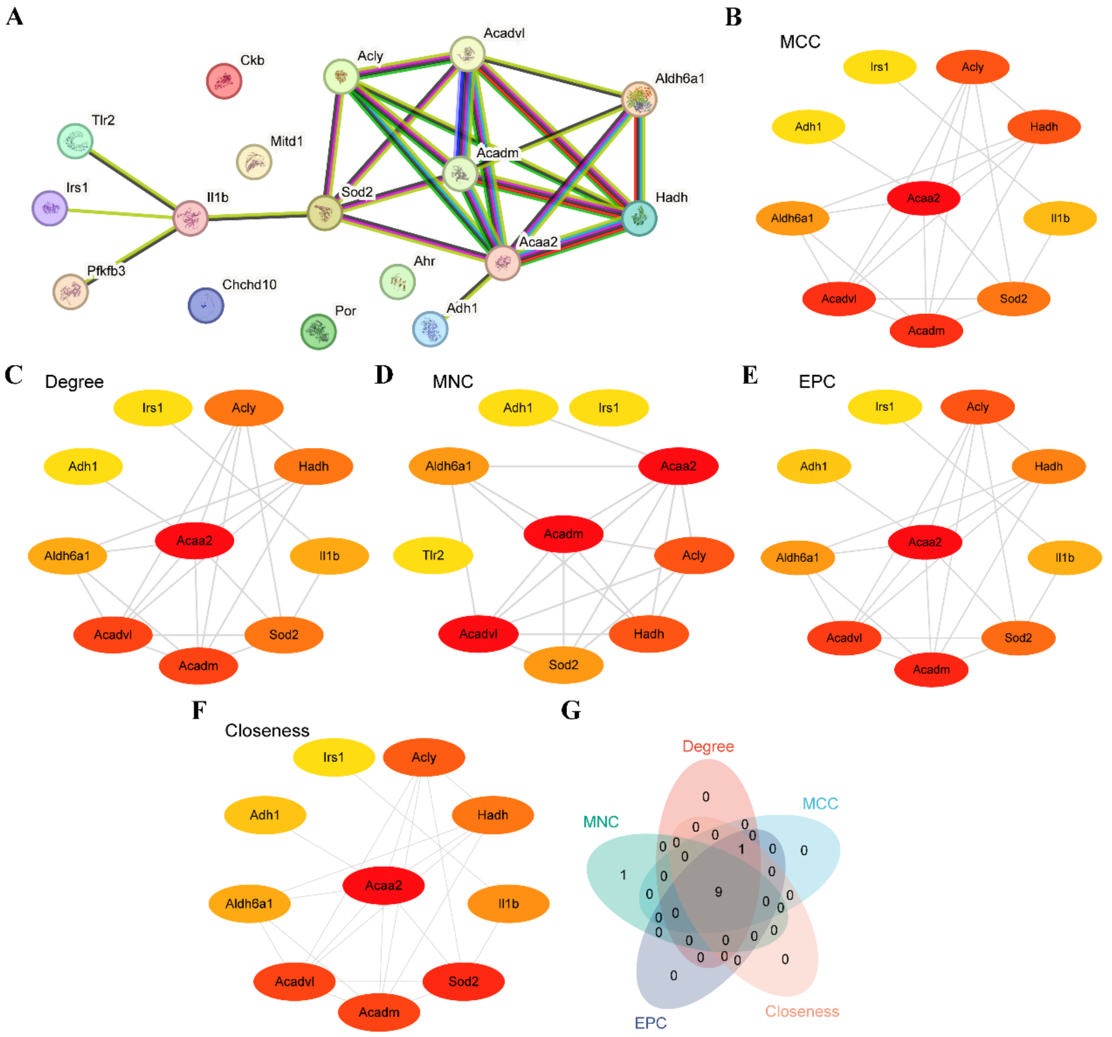

Fig. S1. PPI Network and Hub Genes Analysis.

A. PPI networkn of MEMRDEGs calculated from STRING database; B-F. PPI network of TOP10 MEMRDEGs calculated by 5 algorithms of the CytoHubba plug-in, including MCC (B), MNC (C), Degree (D), EPC (E) and Closeness (F); G. Venn diagram of MEMRDEGs of TOP10 for the 5 algorithms of the CytoHubba plugin. PPI network, Protein-protein Interaction Network; MEMRDEGs, Mitochondrial Energy Metabolism-Related Differentially Expressed Genes; MCC, Maximal Clique Centrality; MNC, Maximum Neighborhood Component; EPC, Edge Percolated Component.

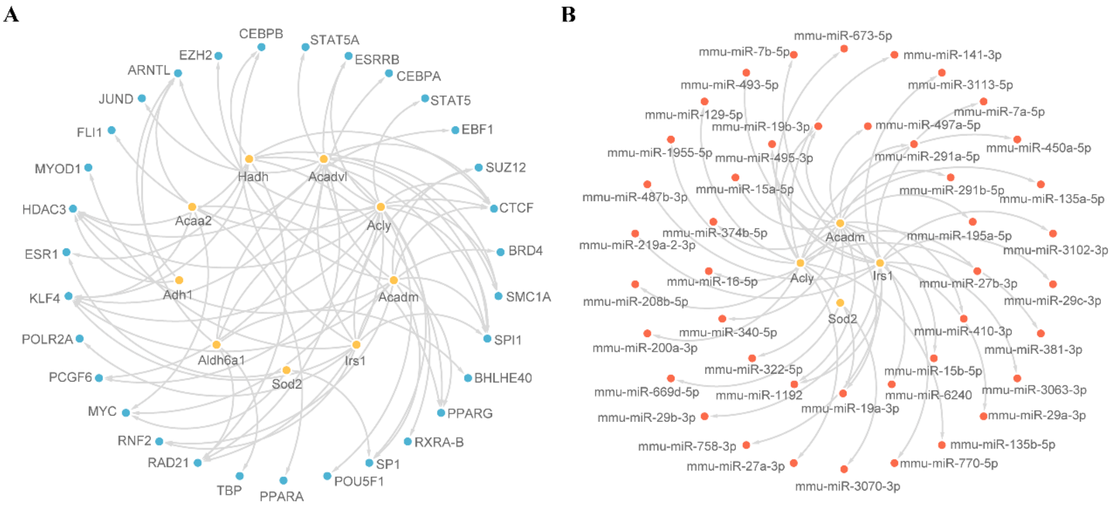

Fig. S2. Regulatory Network of Hub Genes.

A. mRNA-TF Regulatory Network of Hub Genes; B. mRNA-miRNA Regulatory Network of Hub Genes. TF, Transcription Factor. miRNAs are shown in yellow, transcription factors (TFs) in blue, and miRNAs in red.
